# Partial Genetic Characterization of Peste Des Petits Ruminants Virus from Goats in Northern and Eastern Tanzania

**DOI:** 10.1111/tbed.12229

**Published:** 2014-08-19

**Authors:** T Kgotlele, E S Macha, C J Kasanga, L J M Kusiluka, E D Karimuribo, J Van Doorsselaere, J J Wensman, M Munir, G Misinzo

**Affiliations:** 1 Department of Veterinary Microbiology and Parasitology, Faculty of Veterinary Medicine, Sokoine University of AgricultureMorogoro, Tanzania; 2 Department of Veterinary Medicine and Public Health, Faculty of Veterinary Medicine, Sokoine University of AgricultureMorogoro, Tanzania; 3 Higher Institute for Nursing and Biotechnology, VIVES University CollegeRoeselare, Belgium; 4 Department of Clinical Sciences, Swedish University of Agricultural SciencesUppsala, Sweden; 5 The Pirbright Institute, Compton LaboratoryNewbury, Berkshire, UK

**Keywords:** peste des petits ruminants, peste des petits ruminants virus, phylogeny, nucleoprotein gene, Tanzania

## Abstract

Peste des petits ruminants (PPR) is an acute viral disease of small ruminants. The disease was first reported in Tanzania in 2008 when it was confined to the Northern Zone districts bordering Kenya. The present study was carried out to confirm the presence of PPR virus (PPRV) in Tanzania and to establish their phylogenetic relationships. Samples (oculonasal swabs, tissues and whole blood) were obtained from live goats with clinical presentation suggestive of PPR and goats that died naturally in Ngorongoro (Northern Tanzania) and Mvomero (Eastern Tanzania) districts. The clinical signs observed in goats suspected with PPR included fever, dullness, diarrhea, lacrimation, matting of eye lids, purulent oculonasal discharges, cutaneous nodules, erosions on the soft palate and gums and labored breathing. Post mortem findings included pneumonia, congestion of the intestines, and hemorrhages in lymph nodes associated with the respiratory and gastrointestinal systems. PPRV was detected in 21 out of 71 tested animals using primers targeting the nucleoprotein (N) gene. Phylogenetic analysis, based on the N gene, indicated that PPRV obtained from Northern and Eastern Tanzania clustered with PPRV strains of Lineage III, together with PPRV from Sudan and Ethiopia. The findings of this study indicate that there are active PPRV infections in Northern and Eastern Tanzania, suggesting risks for potential spread of PPR in the rest of Tanzania.

## Introduction

Peste des petits ruminants (PPR) is a highly contagious viral disease of wild and domestic small ruminants and camels characterized by oculonasal discharge, stomatitis, diarrhea and pneumonia (Kulkarni et al., 1996; Munir et al., 2013). It is a disease of economic significance because of its transboundary nature, high morbidity and mortality which result in loss of production, abortion, death, limits on export and threat to human food chain (Zahur et al., 2011). The disease is caused by a virus belonging to the genus *Morbillivirus* of the family *Paramyxoviridae*. The PPR virus (PPRV) is highly contagious and easily transmitted by direct contact through the secretions and/or excretions of infected animals to nearby healthy animals (Banyard et al., 2010; Munir et al., 2013). Contact and movement of animals from affected to unaffected areas play an important role in transmitting PPRV especially where communal grazing system is practiced.

The disease was first reported in West Africa in the early 1940s and later recognized as endemic in both West and Central Africa (Abraham et al., 2005). Currently, PPR is prevalent in Central, Eastern and Western Africa, Asia, and the Near and Middle East (Geerts, 2009; Banyard et al., 2010; Munir et al., 2012a, b, 2013). PPRV has been classified into four lineages that are distinct to different geographical locations based on variable nucleotide sequences of the nucleoprotein (N) gene. Western and Central African PPRV cluster into Lineages I and II, Eastern African and PPRV found in the southern part of Middle East cluster into Lineage III while Asian PPRV mainly cluster into Lineage IV (Munir et al., 2013).

Wambura (2000) confirmed the absence of PPR antibodies in the first comprehensive serological study done in 1998 to determine the status of PPR among Tanzanian sheep and goats. However, subsequent studies confirmed the presence of PPR antibodies in goats and sheep of Northern Tanzania in 2004 (Karimuribo et al., 2011), in 2009 (Swai et al., 2009) and between 2008 and 2010 (Kivaria et al., 2013). Antibodies against PPRV have been detected in cattle sampled in 2011 in Northern Tanzania within the Loliondo Controlled Game Area (Lembo et al., 2013). In 2011, an outbreak of PPR in goats and sheep was reported and confirmed in Southern Tanzania (Muse et al., 2012), however, limited data is available about the disease in the rest of Tanzania. The objective of this study was to determine presence of active PPRV infections in Eastern and Northern Tanzania and establish the phylogenetic relationship between Tanzanian and previously characterized PPRV isolates.

## Materials and Methods

### Study area

**Fig 1:**
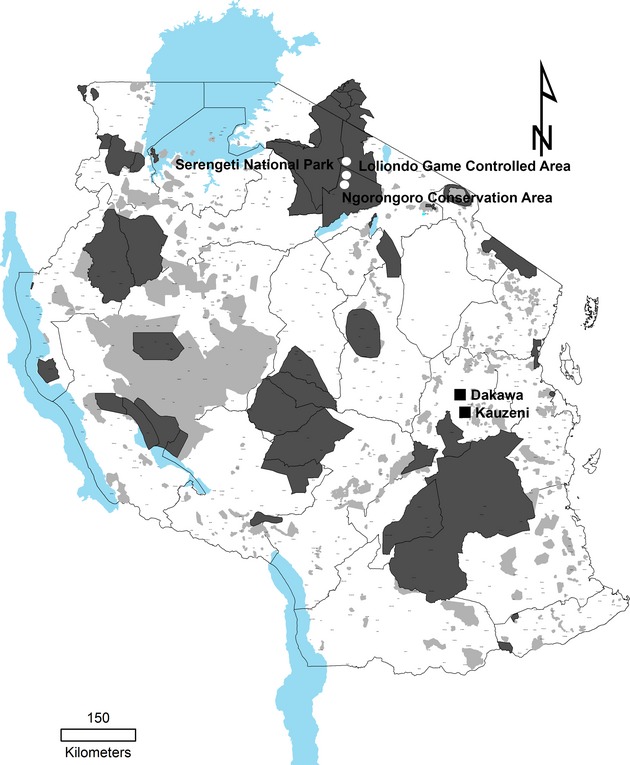
Sampling sites for peste des petits ruminants virus (PPRV). A map of Tanzania showing areas where samples for PPRV were obtained from goats in (i) Northern Tanzania at villages (indicated by white circles) bordering the Serengeti National Park within the Ngorongoro Conservation Area and Loliondo Game Controlled Area and in Eastern Tanzania at Dakawa and Kauzeni villages (indicated by squares) within Mvomero district.

The present study was conducted in Ngorongoro district in Arusha region located in Northern Tanzania and Mvomero district in Morogoro region located in Eastern Tanzania. The Ngorongoro district was selected owing to occurrence of several PPR outbreaks since 2008 even in the areas where vaccination was practiced. The District is considered to be a risk area because it borders Kenya, from which it is believed that the disease was introduced into Northern Tanzania (Kivaria et al., 2013) and it forms part of the Northern transboundary animal movement route. The Mvomero district was also included in the present study because farmers reported outbreaks of a disease resembling PPR. In Mvomero district, samples were collected from goats in November 2012 and January 2013 in Kauzeni and Dakawa villages, respectively (Fig. 1). In Ngorongoro district, samples were obtained in February and March 2013 from villages bordering the Serengeti National Park including Meshili village within Ngorongoro Conservation Area and Piyaya and Malambo villages within Loliondo Game Controlled Area (Fig. 1). In both Mvomero and Ngorongoro districts, samples were obtained from goats belonging to maasai pastoralists.

### Sample collection and processing

Nasal and ocular swabs, and whole blood samples were obtained from randomly selected live goats with clinical presentation suggestive of PPR. Whole blood was collected from the jugular vein using EDTA-containing vacutainer tubes (BD Biosciences, Franklin Lakes, NJ, USA). In addition, lungs, intestines, lymph nodes, whole blood and, ocular and nasal swabs were obtained from five goats in Mvomero district and four goats in Ngorongoro district that died naturally. Buffy coat from whole blood was obtained by centrifuging 500 μl of histopaque (Sigma-Aldrich, St. Louis, MO, USA) layered with 1 ml of whole blood at 400 ***g*** for 30 min at 4°C. The upper layer was discarded while the opaque interface containing the buffy coat was carefully collected. Nasal and ocular swabs were collected and placed in universal viral transport medium (BD Biosciences) followed by vortexing to dislodge cells from the swabs and centrifugation at 8000 ***g*** for 5 min at room temperature. Portions of lungs, intestines and lymph nodes from the same animal were pooled before they were homogenized in F-12 basal cell culture medium (Invitrogen, Carlsbad, CA, USA) to obtain a 10% tissue suspension. The tissue supernatant was obtained by centrifugation of tissue suspensions at 8000 ***g*** for 5 min at room temperature. All samples and the buffy coat were stored at −80°C until RNA extraction was performed.

### PPRV RNA extraction and amplification of PPRV nucleoprotein gene

Viral RNA was recovered from buffy coat, swabs and homogenized tissue samples using a commercial QIAamp viral RNA kit (Qiagen, Hilden, Germany) according to the manufacturer's instructions. PPRV nucleoprotein (N) gene was amplified in a 25 μl reaction using AgPath-ID One-Step RT-PCR kit (Applied Biosystems, Courtaboeuf, France) using PPRV-specific primers NP3 (5′-TCTCGGAAATCGCCTCACAGACTG-3′) and NP4 (5′-CCTCCTCCTGGTCCTCCAGAATCT-3′) as previously described by Couacy-Hymann et al. (2002). Briefly, a reverse transcription was carried out at 45°C for 30 min before initial denaturation of DNA at 95°C followed by 40 cycles of denaturation at 95°C for 30 s, annealing at 55°C for 30 s and elongation at 72°C for 30 s, and a final extension at 72°C for 10 min. Amplification was carried out in a GeneAmp PCR System 9700 (Applied Biosystems, Foster City, CA, USA) followed by electrophoresis on a 1.5% agarose gel. Agarose was pre-mixed with GelRed nucleic acid stain (Phenix Research Products, Candler, NC, USA) and visualization of PCR products was done using a BioDoc-It imaging system (UVP, Upland, CA, USA). PCR products were treated with exonuclease I and alkaline phosphatase before they were sequenced directly using Big Dye Terminator Cycle Sequencing Kit Version 3.1 (Applied Biosystems). Alternatively, PCR fragments were purified from agarose gels using a NucleoSpin gel and PCR clean-up kit (Macherey-Nagel, Düren, Germany). The gel purified PCR fragments were cloned in a TOPO-T/A plasmid vector (pCR4-TOPO) (Invitrogen). Afterwards, dideoxynucleotide cycle sequencing reaction was performed using standard M13 forward (5′-GTAAAACGACGGCCAG-3′) and M13 reverse (5′-CAGGAAACAGCTATGAC-3′) primers for sequencing of PPRV partial N gene inserts in the TOPO-T/A plasmid vectors using Big Dye Terminator Cycle Sequencing Kit Version 3.1 (Applied Biosystems). Sequencing PCR products were purified by ethanol precipitation and separated on a 3500 Genetic Analyzer (Applied Biosystems).

### Phylogenetic relationship of PPRV N gene sequences

The forward and the reverse complement nucleotide sequences delimited by forward and reverse primers of several N gene PCR products of PPRV from the same location were aligned to obtain a consensus nucleotide sequence. A set of sequences representing all lineages of PPRV, including newly reported PPRV sequences from African countries, was used for phylogenetic analysis. Sequences were aligned using ClustalW algorithm in BioEdit (Ibis Biosciences, Carlsbad, CA, USA) and clustering pattern was determined by neighbor-joining method using the Kimura-2-parameter option implemented within mega 5 (Tamura et al., 2011).

## Results

### Clinical signs and post mortem findings observed in goats suspected with PPR

**Fig 2:**
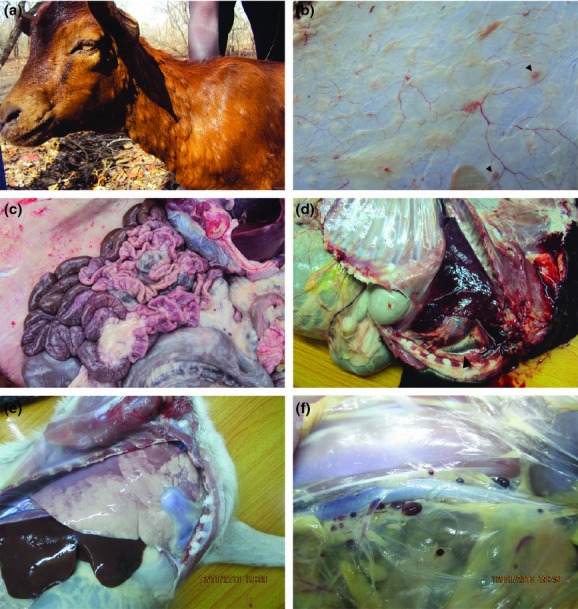
Clinical signs and post mortem findings in goats with peste des petits ruminants (PPR). (a) A goat with oculonasal discharges, periorbital edema and cutaneous nodules. (b) After skinning, cutaneous nodules were mainly confined within the skin with the exception of a few cutaneous nodules that could be observed below the skin (arrow head). Other postmorterm findings in goats with PPR included congestion of intestines (c), pneumonia (d and e) and froth formation (d, arrow head) and hemorrhage of the lymph nodes draining internal organs within thoracic and abdominal cavities (f).

The main clinical signs observed in goats suspected with PPR included fever, dullness, diarrhea, lacrimation, matting of eye lids, purulent oculonasal discharges, cutaneous nodules, erosions on the soft palate and gums and laboured breathing. In some animals, raised cutaneous nodules of up to 3 cm in size were observed especially on the neck region (Fig. 2). The rectal temperature of the examined goats ranged between 39.1 to 41°C, with most animals examined having temperatures above 40°C. Post mortem findings included pneumonia, dark red and firm to touch areas in the lungs, haemorrhages and congestion of the intestines, watery intestinal contents and hemorrhages of lymph nodes associated with the respiratory and gastrointestinal systems (Fig. 2).

### Detection of PPRV by reverse transcription polymerase chain reaction (RT-PCR)

Clinical samples obtained from goats suspected of PPR were tested for the presence of PPRV by RT-PCR targeting the N gene using primers NP3 and NP4. Tested samples were scored positive if a PCR product of approximately 300 bp was visualized after agarose gel electrophoresis. Of the tested goats, 31.1% (*n* = 45) and 26.9% (*n* = 26) were positive for PPRV N gene in Mvomero and Ngorongoro districts, respectively. The rate of detection of PPRV was higher in tissues than in buffy coat, and ocular and nasal swabs (Table 1).

**Table 1:** Distribution of PPRV based on detected of the N gene using reverse transcription polymerase chain reaction (RT-PCR) assay in goat samples collected from Mvomero district located in eastern Tanzania and Ngorongoro district located in northern Tanzania. Depending on availability, more than one sample types from a single animal were tested

Location	Sample type	Positive	Negative	Total	Positive (%)
Mvomero	Swabs	8	23	31	26.0
	Whole blood	2	8	10	20.0
	Tissues	5	0	5	100.0
	Animals	14	31	45	31.1
Ngorongoro	Swabs	2	10	12	16.7
	Whole blood	3	7	10	30.0
	Tissues	2	4	6	33.3
	Animals	7	19	26	26.9

### Phylogenetic relationship of PPRV

**Fig 3:**
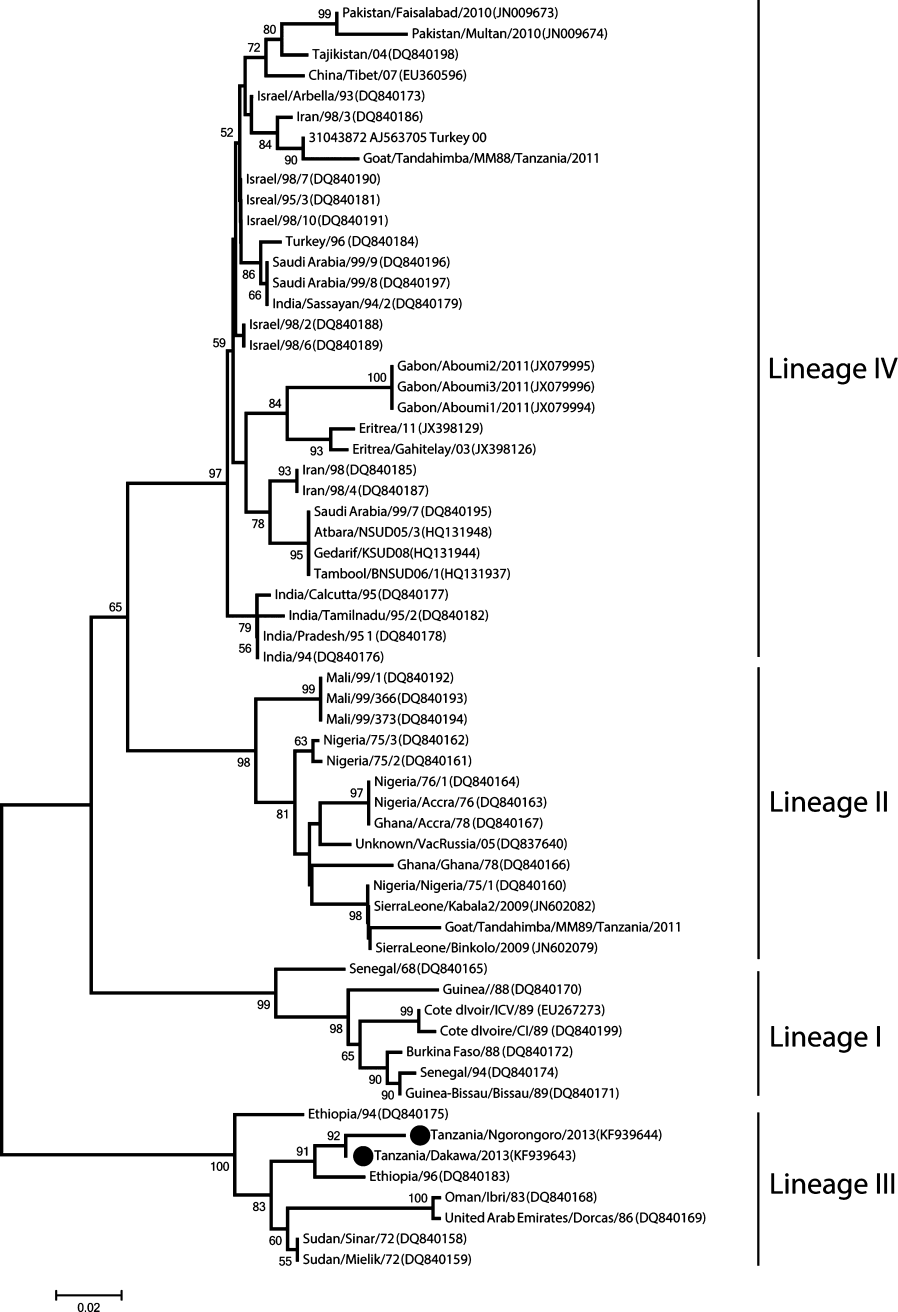
Phylogenetic relationship of peste des petits ruminants viruses (PPRV). A neighbour-joining phylogenetic tree depicting the relationship of Tanzanian PPRV obtained from this study (indicated with circles) with other PPRV belonging to Lineages I-IV. Tanzanian PPRV from Northern Tanzania (Ngorongoro) and Eastern Tanzania (Dakawa) are not 100% identical and are clustered within Lineages III. Phylogeny was inferred following 1000 bootstrap replications and values <50% were not shown.

The N gene nucleotide sequences of Tanzanian PPRV obtained from this study were submitted at GenBank and provided with accession numbers KF939643 and KF939644 for Tanzania/Dakawa/2013 and Tanzania/Ngorongoro/2013 strains, respectively. Alignment of the N gene nucleotide sequences of PPRV obtained from Mvomero showed that the sequences were 100% identical. Similarly, the N gene nucleotide sequences of PPRV obtained from positive animals in Ngorongoro were found to be 100% identical. Therefore, single sequence from each area was selected for phylogenetic analysis. However, the 320 nucleotides long N gene sequences of PPRV from Mvomero and Ngorongoro had two nucleotide substitutions. Translation of the sequences showed that one nucleotide substitution was nonsense while the other substitution led to a change of an amino acid at position 60 (G→A). BLASTN analysis of both Dakawa and Ngorongoro N gene nucleotide sequences of PPRV showed 96% identity to PPRV from Sudan and Ethiopia. A phylogenetic tree based on N gene nucleotide sequences was constructed using the PPRV N gene sequences obtained in this study and other viruses representing the four PPRV lineages. The N gene sequences of PPRV from Mvomero and Ngorongoro districts clustered into Lineage III together with other PPRV previously reported from Ethiopia, Sudan, Oman and United Arab Emirates (Fig. 3).

## Discussion

The circulation of PPRV in selected parts of Eastern and Northern Tanzania was investigated in the present study. The results obtained show that PPRV is circulating in goats in Mvomero and Ngorongoro districts as confirmed by molecular detection of PPRV genome, clinical signs and post-mortem findings in goats. Furthermore, molecular characterization of PPRV based on partial amplification of the N gene show that PPRV, belonging to Lineage III, is circulating both in Eastern and Northern Tanzania. Previous studies have confirmed the presence of PPR in Northern Tanzania based on serological detection of antibodies against PPRV in goats, sheep and cattle (Swai et al., 2009; Karimuribo et al., 2011; Kivaria et al., 2013; Lembo et al., 2013). However, PPR has not been detected in wild small ruminants in Tanzania neither on molecular nor serological diagnostic bases (Lembo et al., 2013).

The presence of PPRV in Southern Tanzania based on PPRV genome detection using RT-PCR assay has been confirmed (Muse et al., 2012). Kivaria et al. (2013) sampled goats and sheep between 2008 and 2010 in Northern Tanzania and reported the circulation of PPRV belonging to lineage III. The present study reports the continued circulation of lineage III PPRV in Northern and its incursion in Eastern Tanzania. PPR is believed to have crossed into Tanzania through Northern Tanzania in 2008 (Swai et al., 2009; Karimuribo et al., 2011). As PPRV obtained from the present study cluster with PPRV from Sudan and Ethiopia, it is reasonable to assume that probably, PPRV crossed into Northern Tanzania from these countries probably through Kenya (Kivaria et al., 2013). It is less likely that PPRV crossed into Tanzania through Uganda, as PPRV lineage I, II and IV but not lineage III have been reported in Uganda (Luka et al., 2012b).

Afterwards, it is believed that PPRV spread to other parts of the country from Northern Tanzania (Muse et al., 2012; Kivaria et al., 2013). The results obtained from the present study may indicate that PPRV has spread from Northern to the Eastern Tanzania because of the high genetic identity of the variable N gene. Further in-depth studies are required to elucidate the origin and transmission characteristics of PPRV in Tanzania and neighboring countries. Previous reports showed high seroprevalence of PPR (45.4%) in Northern Tanzania (Swai et al., 2009) and PPRV infection of 31.0% in Southern Tanzania (Muse et al., 2012). In the present study, PPRV genome was detected in 29.6% and 31.1% of the goats tested in Northern and Eastern Tanzania, respectively. However, this may not be a true reflection of the PPRV prevalence because only goats showing PPR clinical signs were sampled in the present study. PPRV could not be detected in all of the goats tested despite the fact that samples were collected from animals showing PPR clinical presentation. Previous studies have shown that the type of sample used during diagnosis of PPR, stage of infection and the type of gene targeted for RT-PCR may influence the level of positivity (Luka et al., 2012a). In the present study, a high proportion of positive animals were detected when tissue samples were used in the diagnosis of PPR. However, PPRV was detected from various samples including oculonasal swabs, buffy coat and tissues (lymph nodes, intestines and lungs). The ability to detect PPRV in oculonasal swabs is an important finding because swabs taken from infected animals in the field not only provide a suitable source of viral RNA but also are not subject to the same storage and transport problems associated with tissue samples (Forsyth and Barrett, 1995).

Little success on preventing active PPRV infections has been achieved in Ngorongoro district despite multiple vaccination campaigns in the district. Probably PPRV active infections cross from wildlife because domestic animals interact with wildlife in Ngorongoro Conservation Area and the Loliondo Game Controlled Area. However such link has not clearly been established yet (Munir, 2013), which requires future extensive studies to understand the possible spread between domestic and wild small ruminants. In addition, there is high and frequent movement of goats between Tanzania and Kenya along the Northern border for trade. Trade of live animals at markets has been shown to be an important vehicle for transmission of infectious diseases (Domenech et al., 2006). The role of animal markets in the transmission of PPR was also reported by Muse et al. (2012), who found that inadequate infrastructure especially in local animal markets may be facilitating transmission of PPR.

Taken together, we conclude that an active PPRV infection is present in Northern and Eastern Tanzania. The characterized strains of PPRV belong to Lineage III and are different from the Nig75/1 (Lineage II) currently being used as vaccine in the region.
